# The Association of Hospital Matrons

**Published:** 1921-07-16

**Authors:** 


### The Association of Hospital Matrons.

Despite the intensity of the heat on Saturday, July 9, a goodly number of matrons attended the annual meeting of the Association of Hospital Matrons held that afternoon, by kind permission of the Treasurer and Miss Lloyd Still, in the Great Hall of St. Thomas's Hospital. Amongst those who came from the provinces were Miss Cummins (Liverpool), Miss Steele (York), Miss Cann (Norwich), Miss Sparshott (Manchester), Miss Sutcliffe (Derby), Miss Carpenter Turner (Winchester), and Miss Vickers (Yorkshire). The Association has steadily progressed during the past year; seventy-four new members have been enrolled, and two new groups have been formed—*i.e.*, Yorkshire and Liverpool. The influence and usefulness of these local groups are evident from the reports of each which were submitted to the annual meeting of the parent body. Subjects of paramount interest have been discussed and expression given to the views and difficulties of matrons working in different parts of the country in institutions large or small and with varying conditions. The total membership of the Association is 426, and the members come from eighty-three general hospitals of over 100 beds, 70 of under 100 beds, 129 special hospitals, 38 Poor-law infirmaries, 19 military hospitals, and 58 nursing institutes; 29 are late matrons. The expenditure for the year was £118, and the income £181, leaving with the amount brought forward a credit balance of £101. The Sale of Work produced £71 18s., an increase of £10 on the previous year. It is from this source that the expenses of local delegates to attend the quarterly meetings are paid, and Miss Lloyd Still, President of the Association, expressed the hope that at the quarterly meeting at Leeds, provisionally fixed for November 12, the Sale of Work will prove even a greater success, for the more delegates that can attend these quarterly meetings the better. It is by such visits as these that the spirit of unity can be fostered, and any tendency to a mechanical organisation and atmosphere can be countered. In her opening speech Miss Lloyd Still laid stress on this latter point, and the necessity to guard the profession from becoming too much of an organised machine.

The members of the Council for the coming year elected by ballot are Miss Bailey, Miss Barton, Miss Coulton, and Miss Cummins (all re-elected), Miss Innes, and Miss Wilcox. Great regret was felt and expressed by the President at the absence from the meeting of the Hon. Secretary, Miss Cox-Davies, owing to her illness. The meeting passed a vote of sympathy, and Miss Lloyd Still was able to assure it that Miss Cox-Davies was making satisfactory progress. An interesting discussion on the Draft Syllabus was opened by Miss Cummins, and supported by a paper by Miss Billing, which was read in her absence by Miss McIntosh, who acted as Hon. Secretary in Miss Cox-Davies' place. Miss Hogg and Miss Steele spoke on "Salaries and Hours of Work." At the conclusion of the meeting Miss Lloyd Still entertained the gathering to tea.

Prior to the meeting a service for the members was held in the hospital chapel, at which the Archdeacon of London delivered an address.

